# Pedestrian injury and the built environment: an environmental scan of hotspots

**DOI:** 10.1186/1471-2458-9-233

**Published:** 2009-07-14

**Authors:** Nadine Schuurman, Jonathan Cinnamon, Valorie A Crooks, S Morad Hameed

**Affiliations:** 1 Department of Geography, Simon Fraser University, Burnaby, Canada; 2 Department of Surgery, University of British Columbia, Vancouver, Canada

## Abstract

**Background:**

Pedestrian injury frequently results in devastating and costly injuries and accounts for 11% of all road user fatalities. In the United States in 2006 there were 4,784 fatalities and 61,000 injuries from pedestrian injury, and in 2007 there were 4,654 fatalities and 70,000 injuries. In Canada, injury is the leading cause of death for those under 45 years of age and the fourth most common cause of death for all ages Traumatic pedestrian injury results in nearly 4000 hospitalizations in Canada annually. These injuries result from the interplay of modifiable environmental factors. The objective of this study was to determine links between the built environment and pedestrian injury hotspots in Vancouver.

**Methods:**

Data were obtained from the Insurance Corporation of British Columbia (ICBC) for the 6 year period from 2000 to 2005 and combined with pedestrian injury data extracted from the British Columbia Trauma Registry (BCTR) for the same period. High incident locations (hotspots) for pedestrian injury in the City of Vancouver were identified and mapped using geographic information systems (GIS), and the characteristics of the built environment at each of the hotspot locations were examined by a team of researchers.

**Results:**

The analysis highlighted 32 pedestrian injury hotspot locations in Vancouver. 31 of 32 hotspots were situated on major roads. Likewise, the majority of hotspots were located on downtown streets. The 'downtown eastside' was identified as an area with multiple high-incident locations, including the 2 highest ranked pedestrian injury hotspots. Bars were present at 21 of the hotspot locations, with 11 of these locations being judged to have high alcohol establishment density.

**Conclusion:**

This study highlighted the disproportionate burden of pedestrian injury centred on the downtown eastside area of Vancouver. The environmental scan revealed that important passive pedestrian safety countermeasures were only present at a minority of high-incident locations. More importantly, bars were highly associated with risk of pedestrian injury. This study is the basis for potential public health intervention by clearly indicating optimal locations for signalized pedestrian crosswalks.

## Background

The World Health Organization [1] estimates that more than five million people around the world die annually as a result of injury. Half-a-million are in high-income countries alone, where they account for 6% of all deaths. In high-income countries, road traffic injuries (including pedestrian trauma), self-inflicted injuries and interpersonal violence are the three leading causes of death among people aged 15–29 years [2]. In Canada, injury is the leading cause of death for those under 45 years of age and the fourth most common cause of death for all ages. Collisions between motor-vehicles and pedestrians claim hundreds of lives and injure tens of thousands annually [3]. Traumatic pedestrian injury, in particular, results in around 4000 hospitalizations in Canada each year [4]. These injuries often result from the interplay of modifiable or preventable environmental factors [5]. Addressing the environmental factors related to pedestrian injury thus represents an important public health opportunity.

Active interventions to reduce the toll of pedestrian injury centre on educating drivers and pedestrians in road safety and enforcement of traffic safety laws, while passive interventions largely involve modifications to the built-environment [6,7]. Designing pedestrian-friendly roadways has the potential to reduce pedestrian injury [7,8]; however the movement of motorized vehicles remains the primary design objective for road engineers, while pedestrian safety is often an afterthought [9,10]. Research has linked aspects of the built environment, roadway infrastructure, and types of land-use to an increase or decrease in the risk of pedestrian injury. For example, roadway design factors including curb parking, long blocks, and the absence of marked and signalized crosswalks are associated with an increase in the risk of collisions between pedestrians and vehicles. Certain types of land uses have been linked with increases in pedestrian injury incidence, in particular schools and alcohol serving establishments [11-22]. Lower vehicle speeds, exclusive turn phasing at intersections, and medians have been shown to reduce pedestrian-vehicle encounters [8]. These and other environmental countermeasures are highly-effective, low-cost solutions that can be implemented at high-risk sites to help reduce the burden of pedestrian injury [23].

Geographic Information Systems (GIS) are a valuable tool for epidemiological research [24]. A handful of pedestrian injury studies have used GIS to analyze incident locations; however, few of these studies include a comprehensive analysis of the environmental factors that may be contributing to the risk of pedestrian injury [25-27]. The goals of this study were (i) to use GIS to determine pedestrian injury hotspot locations in the City of Vancouver and (ii) to determine key characteristics of the built-environment that may contribute to increased risk of pedestrian injury. Results of this study highlight important areas of the city that should be targeted for safety interventions, and may be useful for directing strategies to implement environmental countermeasures. In addition, this study introduces an innovative methodological advancement in using GIS-based hot spot analysis in combination with a detailed environmental scan.

## Methods

In this study, high incident locations (hotspots) for pedestrian injury in the City of Vancouver were identified using GIS, and the characteristics of the built environment at each of the hotspot locations were examined. Two pedestrian injury data sources were combined for the analysis: The Insurance Corporation of British Columbia's (ICBC) pedestrian-vehicle collision data for the 6 year period from 2000 to 2005 inclusive, and the British Columbia Trauma Registry's (BCTR) pedestrian injury records for the 6 year period 2001 to 2006 inclusive. The ICBC source records all reported incidents, while the BCTR source records all incidents that resulted in a hospital stay of two days or more. Combined, and with duplicate records removed, data from the two sources can be considered representative of the majority of injuries from pedestrian-vehicle collisions over a given 6 year period. It should be noted that each record involved one person. If a road traffic collision involved two people, then there would be two incidents for that particular hotspot. For the purposes of this study, hotspots were determined to be locations where a minimum of 5 incidents were recorded in both datasets combined. Hotspots were then ranked according to the number of incidents recorded over the 6 year period. More severe incidents were ranked equally with "near misses" recorded as minor injuries. The rationale for this is that these minor incidents were as potentially lethal.

Incident locations were mapped using ArcGIS 9.2, [28] georeferenced to either an intersection or midblock location. The ICBC data were mapped according to latitude and longitude coordinates provided in the dataset for each incident location. The BCTR data were geocoded based on the street address or intersection where the incident occurred which was available in the dataset. The frequency of non-mappable records was negligible, and all data were assumed to be accurate and complete. A kernel density map was created to allow for a simple visual examination of incident locations and precise identification of all hotspots. A kernel search distance of 100 m was used, as it proved to be the most appropriate distance for highlighting unique incident locations. Elements of the built-environment and roadway design were recorded at each of the hotspot locations to examine their potential contribution to pedestrian injury. A team of 4 researchers independently surveyed each hotspot to assess 14 pre-determined built-environment characteristics, and recorded any other particularities observed at each location (e.g., changes in roadway slope). Prior to the observational period the investigators met to discuss how each variable should be interpreted in order to enhance the consistency of recorded data. The four investigators independently completed their observations at each intersection on a standard recording sheet and then immediately met to compare notes and resolve any disagreements or differences in interpretation. Overall there was very little disagreement in the data recorded on the standard sheet across investigators and any that did occur was easily resolved through discussion at the intersection. Finally, all investigators reviewed and agreed with the data shared in the summative table produced (see Table 1).

**Table 1:** Results of the hotspot environmental scan

**LOCATION**	**Incidents**		**Contribute to Risk**					**Complexity**			**Land Use**			**Safety Measures**		
**MIDBLOCK**	**total**	**rank**	**long block**	**bus stop**	**curb park**	**X walk**	**obstruction**	**sign- age**	**# lanes**	**L/R ban**	**bars**	**retail**	**school**	**calming**	**median**	**excl. turn**

E Hastings btw Columbia & Main	49	1	**Y**	**Y**	**Y**	**N**	N	L	6	N/A	**H**	**M**	N	**N**	**N**	N/A
E Hastings btw Jackson & Dunlevy	10	5	**Y**	**Y**	**Y**	**N**	**Y-AD**	L	6	N/A	**M**	**M**	N	**N**	**N**	N/A
E Hastings btw Main & Gore	9	6	**Y**	**Y**	**Y**	**N**	**Y-FL**	L	6	N/A	**H**	**M**	N	**N**	**N**	N/A
Burrard btw Nelson & Comox	8	7	**Y**	**Y**	**Y**	**N**	N	L	6	N/A	N	**L**	N	**N**	**N**	N/A
E Hastings btw Kamloops & Penticton	8	7	**Y**	N	**Y**	**N**	N	L	6	N/A	N	**H**	N	**N**	**N**	N/A
Granville btw Georgia & Dunsmuir	8	7	**Y**	**Y**	N	**N**	**Y-FL**	L	2	N/A	**L**	**H**	N	Y	**N**	N/A
E Hastings btw Heatley & Hawks	7	8	**Y**	N	**Y**	**N**	N	L	6	N/A	**M**	**M**	N	**N**	**N**	N/A
Granville btw Nelson & Helmcken	7	8	N	**Y**	**Y**	**N**	N	L	4	N/A	**H**	**H**	N	**N**	**N**	N/A
Main btw National & Terminal	6	9	N	**Y**	N	**N**	N	L	7	N/A	**H**	**L**	N	**N**	Y	N/A
W Georgia btw Howe & Granville	5	10	N	**Y**	Y*	**N**	**Y-FL**	L	6	N/A	N	N	N	**N**	**N**	N/A
W Hastings btw Carrall & Abbott	5	10	N	N	**Y**	Y	N	L	5	N/A	**H**	**M**	N	**N**	**N**	N/A

**INTERSECTION**																

E Hastings & Main	18	2	**Y**	**Y-A**	**Y**	Y	**Y-FL**	L	12	**N**	**H**	**M**	N	**N**	**N**	Y
E Broadway & Commercial	12	3	**Y**	**Y-B&A**	**Y**	Y	N	M	10	Y	N	**H**	N	**N**	Y	Y
E Broadway & Fraser	12	3	**Y**	**Y-A**	N	Y	N	M	10	**N**	N	**L**	N	**N**	Y	Y
W Georgia & Burrard	12	3	**Y**	**Y-A**	N	Y	N	M	12	Y	N	**H**	N	**N**	**N**	**N**
W Hastings & Carrall	12	3	**Y**	**Y-A**	**Y**	Y	**Y-FL**	L	9	**N**	**H**	**M**	N	**N**	**N**	**N**
E Hastings & Commercial	11	4	**Y**	**Y-B&A**	**Y**	Y	N	M	13	**N**	N	N	N	**N**	Y	Y
E Hastings & Gore	10	5	**Y**	**Y-B&A**	**Y**	Y	N	L	9	**N**	**H**	**M**	N	**N**	**N**	**N**
E 49th & Victoria	9	6	**Y**	**Y-B&A**	N	Y	N	L	8	**N**	N	**M**	N	**N**	**N**	**N**
Howe & Davie	9	6	N	**Y-A**	**Y**	Y	N	**H**	8	**N**	**M**	**L**	N	**N**	**N**	**N**
Commercial & 1st Ave	8	7	N	**Y-A**	**Y**	Y	N	L	13	**N**	**M**	**H**	N	**N**	**N**	Y
E 41st & Fraser	8	7	**Y**	**Y-B&A**	**Y**	Y	N	M	14	**N**	N	**M**	**Y**	**N**	Y	Y
W Broadway & Macdonald	8	7	N	**Y-B&A**	**Y**	Y	**Y-FL**	M	11	**N**	**M**	**H**	N	**N**	**N**	**N**
E Hastings & Renfrew	7	8	**Y**	**Y-B&A**	N	Y	**Y-AD**	**H**	12	**N**	N	N	N	**N**	**N**	Y
Main & Terminal	7	8	**Y**	**Y-B&A**	N	Y	N	L	16	**N**	**L**	**L**	N	**N**	Y	Y
Thurlow & Davie	7	8	N	**Y-A**	**Y**	Y	N	L	6	**N**	**H**	**H**	N	**N**	**N**	**N**
W Hastings & Abbott	7	8	**Y**	**Y-B**	Y	Y	**Y-FL, AD**	L	9	**N**	**H**	**M**	N	**N**	**N**	**N**
Clark & E Broadway	6	9	**Y**	**Y-A**	N	Y	N	L	13	**N**	N	N	**Y**	**N**	Y	Y
E Hastings & Clark	6	9	**Y**	**Y-A**	Y	Y	N	L	12	**N**	**L**	N	N	**N**	Y	Y
Kingsway & E Broadway	6	9	**Y**	**Y-B&A**	**Y**	Y	N	**H**	11	Y	**L**	**H**	N	**N**	Y	Y
Burrard & Davie	5	10	**Y**	**Y-B&A**	**Y**	Y	**Y-FL**	M	10	Y	**H**	**M**	N	**N**	**N**	**N**
W Georgia & Howe	5	10	N	N	Y*	Y	**Y-FL**	M	10	**N**	**L**	**L**	N	**N**	**N**	Y

btw = between, B = before, A = after, FL = flora, AD = advertising, N/A = not applicable, * = illegal, H = high, M = medium, L = low, Y = yes, N = no.

The environmental scan was conducted between 10 am and 3 pm to avoid encountering high traffic volume and associated congestion. Factors that have been shown to increase risk that were included in the assessment were long blocks, presence of bus stops, curb parking, absence of controlled crosswalks, and visual obstructions. Protective factors assessed were the presence of traffic calming measures, medians or pedestrian refuge islands, and exclusive turn signals at intersections. Factors that contribute to location complexity were the number of signs, number of approach lanes, and whether a vehicle turning ban was in effect. Also recorded were the densities of bars, retail establishments, and schools in proximity to the hotspot locations. Privacy was protected both use of the kernel density method and by the clustering of incidents into hotspots of greater than 5 collisions.

Ethics approval for this study was granted from the Office of Research Services at Simon Fraser University (file #37437).

## Results

**Figure 1:**
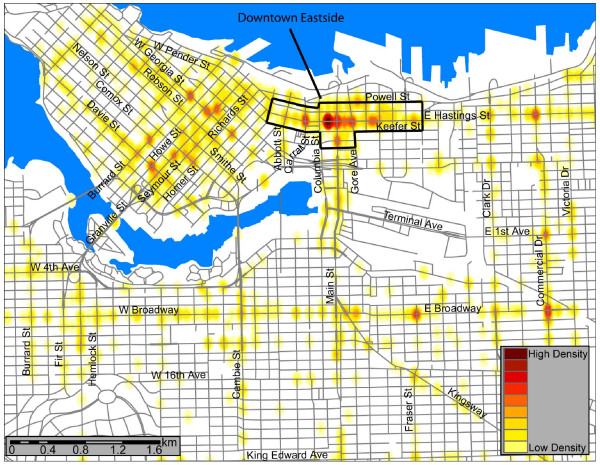
**The intersections and midblock locations where pedestrian injuries were recorded, illuminating the high density locations**.

A total of 2358 pedestrian-vehicle collisions were recorded within the City of Vancouver over the 6 year period, for an average annual pedestrian injury incidence rate of 66.6/100,000 residents. Intersections – rather than midblock locations – accounted for 61% of all incidents. Our analysis highlighted 32 pedestrian injury hotspot locations in the City of Vancouver for this time period. Figure 1 shows the intersections and midblock locations where pedestrian injuries were recorded and illuminates the high density locations. The darkest shades indicate the higher density hotspots. Of the 32 hotspots, 21 (66%) were at intersections while 11 were at midblock locations. Thirty-one of 32 hotspots were situated on either major collector or major arterial roads, with just one at a midblock location of a minor traffic-restricted street. Overall, most hotspots were located on downtown streets. The downtown eastside (DTES) area was particularly highlighted as an area with multiple high-incident locations, including the 2 highest ranked pedestrian injury hotspots. Also of interest is the east-west Broadway corridor – a retail intensive commercial stretch.

Results of the hotspot environmental scan are shown in Table 1. The results of primary interest are highlighted in the table, indicating the presence of demonstrated risk factors for pedestrian injury, or lack of pedestrian safety countermeasures in place at the location. For a majority of midblock and intersection hotspots, long blocks, bus stops, and curb parking were recorded. Only 1 of the 11 midblock locations had a marked and signalized pedestrian crosswalk. Regarding visual obstructions, a minority of locations had advertising or flora which was deemed to be intrusive. Complex signage was observed at just 3 hotspots. The number of approach lanes varied from 2 to 6 for midblock locations with 6 being the most common, and from 6 to 14 for intersections, with 12 the most common. Just 4 of 21 intersections had a turning ban imposed. Almost all (26) locations had retail establishments nearby, with 9 considered to be in high retail density areas. Only 2 locations were situated near schools, and only 1 had traffic-calming measures in place. Nine of 32 hotspots had medians or traffic refuge islands, and 12 of 21 intersections had exclusive turning signals. Bars were present at 21 of the hotspot locations, with 11 of these locations judged to have high alcohol serving establishment density.

## Discussion

The mapping of Vancouver's pedestrian injury hotspots revealed an intriguing spatial pattern. As may be expected, there were more high-incident locations in downtown areas compared with outer areas of the city; however, the disproportionate number of hotspots in a small area of the DTES is conspicuous. Nine of 32 hotspots, and fully 10 per cent of total pedestrian injuries in Vancouver, were recorded within this small part of the downtown core. This area is notorious as the epicentre of homelessness in Vancouver in which large numbers of homeless and other marginalized individuals congregate along Hastings and adjacent streets. It is also where a large number of services aimed at homeless, drug addicted, and/or mentally ill persons are located. It is also likely that the number of alcohol-serving retail establishments within the DTES was a strong factor with respect to the number of hotspots in the area. This is consistent with the existing literature [11-18]. The authors hypothesize that a combination of mental illness, despondency associated with homelessness and high alcohol and substance abuse contributed to the concentration of pedestrian injury in the DTES.

The scan of roadway infrastructure and built-environment characteristics at pedestrian injury hotspots in Vancouver produced two findings of interest. The most striking finding was the frequent presence of demonstrated environmental risk factors, coupled with a scarcity of traffic-calming and passive pedestrian safety countermeasures at many of the high-incident locations. A second important finding from the environmental scan was that bars were closely situated to many of the hotspots.

### The Absence of Pedestrian Safety Countermeasures

**Figure 2:**
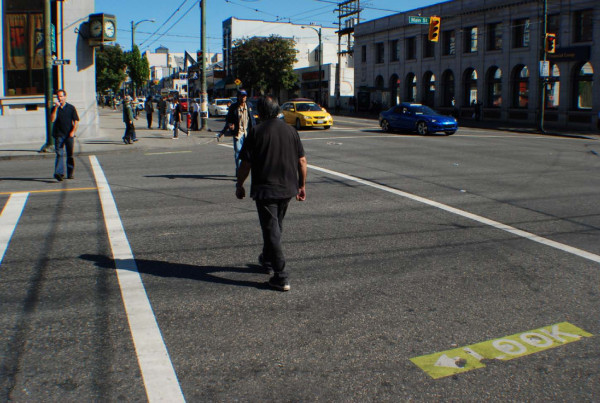
**Intersection at Hastings and Main Streets**.

**Figure 3:**
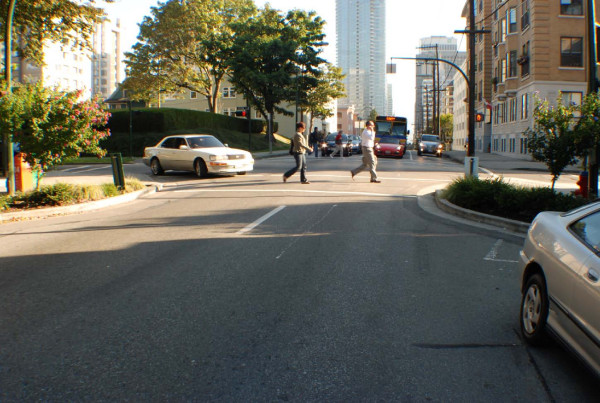
**A location with a corner bulge in place**.

Road safety research has highlighted the influential roles that road infrastructure and the local environment at collision sites contribute to the occurrence of pedestrian injury [29]. Passive safety measures including the development of safe road infrastructure have been successful in reducing the burden of pedestrian injury [30]. Road-dividing medians were absent from a majority of high-incident locations, despite nearly all of the hotspots occurring on major arterial and collector roads. It has been shown that medians or pedestrian refuge islands can reduce pedestrian injury as they promote a two-stage crossing on busy streets and a slight reduction in vehicle speeds [31]. Implementation of medians or refuge islands is likely possible at many of the hotspot locations in which they are absent. The highest-ranked intersection location at Hastings and Main Streets (Figure 2) is a good candidate for installation of a roadway-dividing median which will allow for a two stage crossing if needed, and will likely reduce vehicle speeds in this pedestrian-congested area. Another roadway modification designed with the pedestrian in mind (and endorsed by the City of Vancouver) is corner sidewalk bulges to reduce crossing times for pedestrians [32]. Figure 3 shows a location with a corner bulge in place. Medians and bulges at this hotspot location may require lane narrowing, or a possible lane removal which may result in reduced vehicle flow on this thoroughfare; however, the potential to increase pedestrian safety at this high-incident location should be paramount.

**Figure 4:**
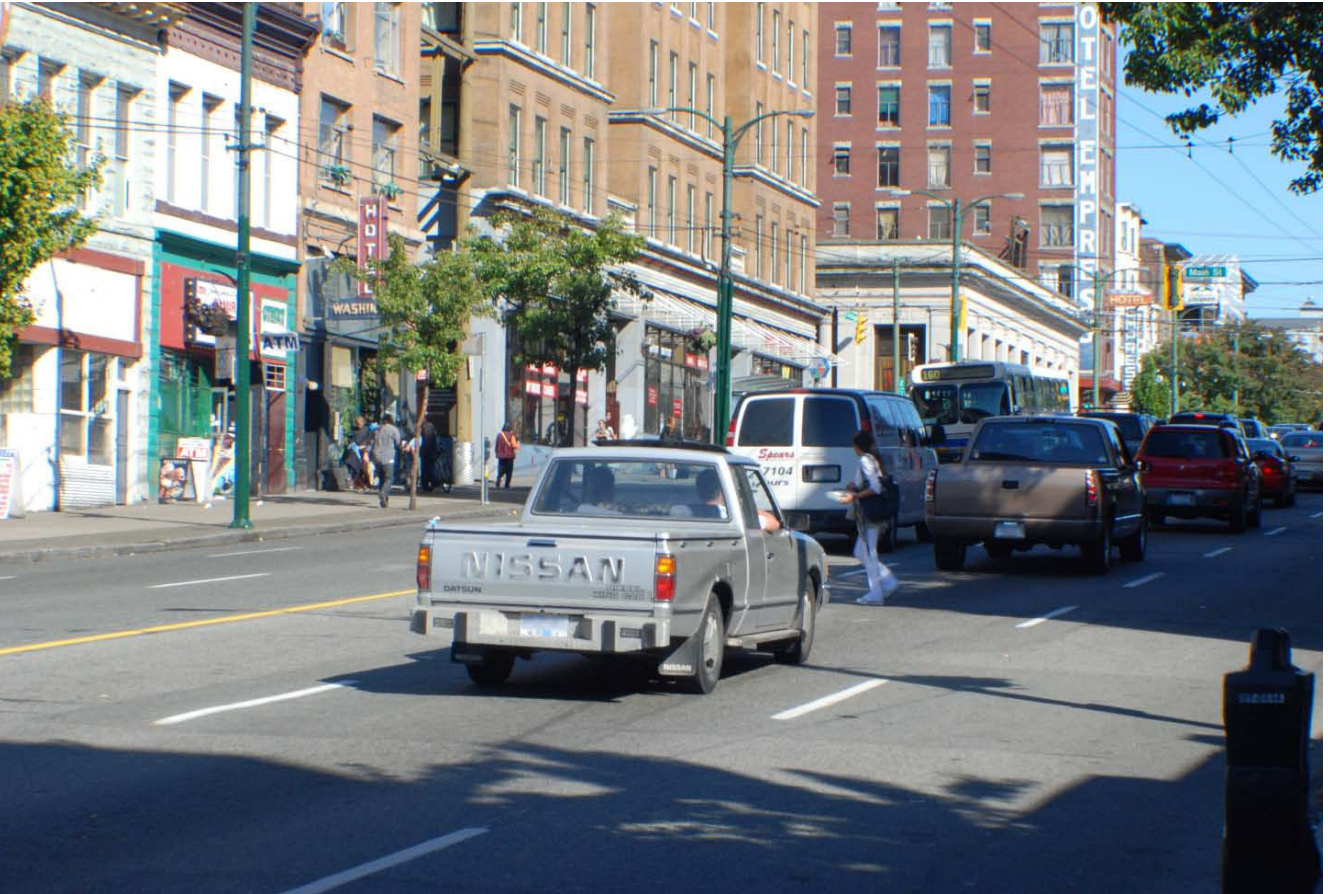
**The midblock location on Hastings St. between Columbia and Main**.

**Figure 5:**
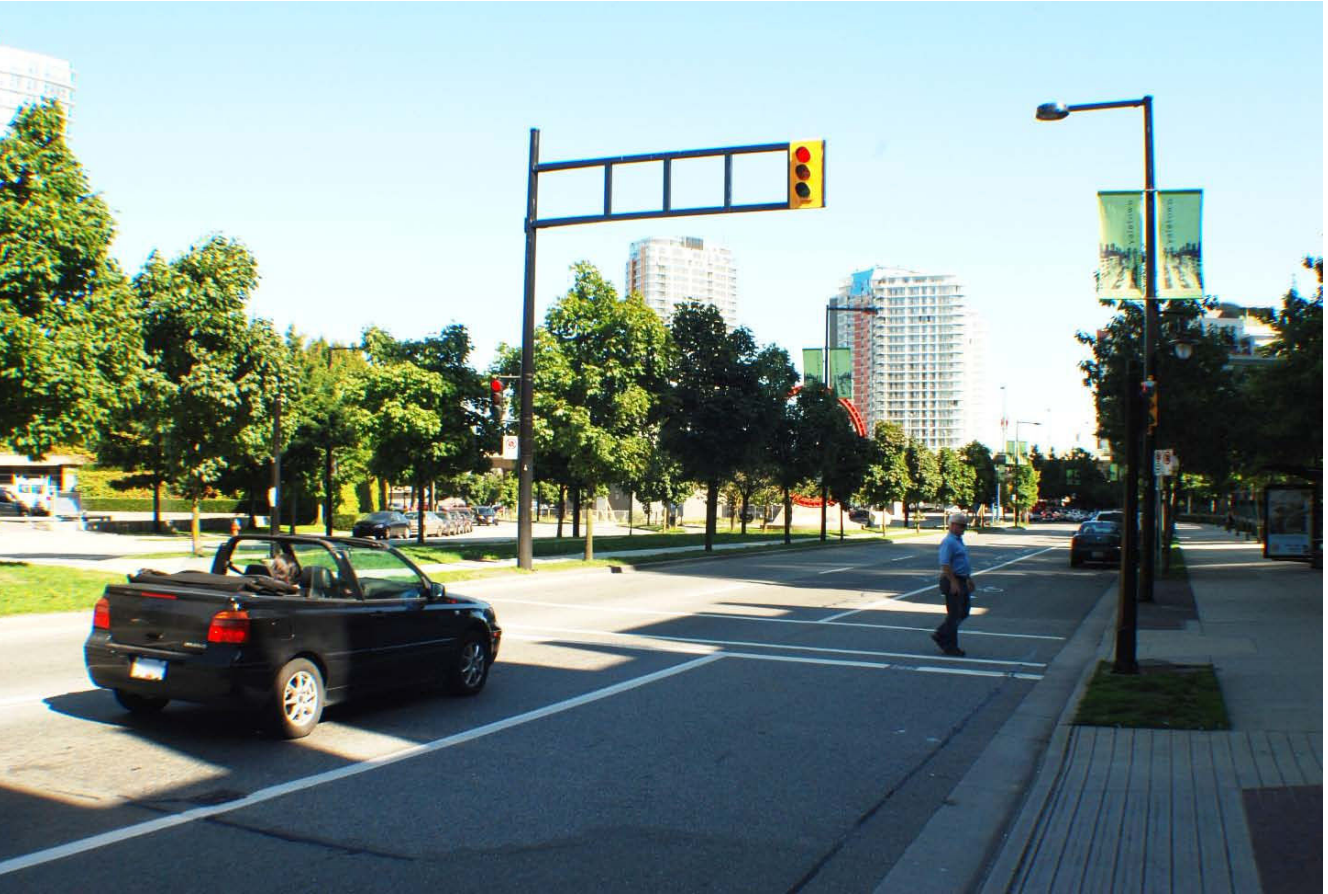
**A signalized crosswalk and median at a midblock location on Expo/Pacific Blvd**.

The absence of marked and signalized pedestrian crossings at all but one midblock location is of particular concern. Well-marked crosswalks with a pedestrian-controlled signal can reduce pedestrian-vehicle conflicts [33]. Another option that has been shown to be effective at midblock locations are non-signalized crosswalks with in-pavement lights that flash when a pedestrian is present [34]. These were not present at any of our hotspot locations. The midblock location on Hastings St. between Columbia and Main was by far the highest ranked incident location (Figure 4). This is one of the main areas of the DTES where large groups of homeless people congregate, and is also the precise location of Insite, the government-sponsored controlled safe drug injection facility. These are likely factors in the disproportionately large number of pedestrian-vehicle collisions at this location; however, no crosswalk, traffic-calming measures, or pedestrian safety interventions are in place at this midblock location. The City of Vancouver has committed to providing midblock crossings on downtown streets near "significant pedestrian generators that create high demands for pedestrian crossing at mid-block" [32]. Figure 5 shows a signalized crosswalk and median at a midblock location on Expo/Pacific Blvd. This type of roadway design/traffic calming measure could potentially improve pedestrian safety at this very high-incident location on Hastings St. Indeed, this study is the basis for concerted and directed intervention on the part of public safety officials. Moreover, it provides a protocol for determining and studying hotspot locations in other cities.

### The Presence of Bars and Alcohol Serving Establishments

Alcohol consumption by pedestrians is a recognized factor influencing their risk of collision with a vehicle; however it is often overlooked as an issue in comparison with alcohol consumption by drivers. Recent consumption of alcohol is common in injured pedestrians, and it has been shown that the severity of injuries is frequently greater for this group [35]. The high incidence of injury in alcohol-affected pedestrians may in part be due to the effects of alcohol on the pedestrian's ability to judge gaps in the traffic for safe road-crossing [36]. Pedestrian injury hotspot locations are often in areas with a high density of bars and other alcohol serving establishments. In a spatial analysis of pedestrian injury in San Francisco, LaScala *et al*. [37] discovered that pedestrian injury was highest in areas with the greatest density of alcohol serving establishments, for incidents where the pedestrian had been consuming alcohol. The results of the present study indicate that bars were located immediately proximal to two-thirds of the hotspots, with almost one-third located in high density bar and alcohol serving establishment areas. Since pedestrian injury patients' alcohol levels are not consistently included in the BCTR, we were unable to gauge whether alcohol was a definitive explanation for the correlation between bars and injuries. However, there is ample reason to suspect that this is the case and policy should proceed accordingly.

Active interventions such as educating the customer and service establishment in safe drinking guidelines have been used to varying success [14]; however, more effective countermeasures may involve modifying the roadway environment or calming traffic to increase the safety of alcohol-affected pedestrians. Results of a study by Lenné *et al*. [38] suggest that modifying traffic signals at high-risk times (late evening and early morning) could help reduce injury in this group. Specifically, if traffic signals in areas of high alcohol establishment density were set to 'dwell-on-red' in all directions when no vehicles were present, then the average speed of vehicles would drop, thus creating a safer pedestrian environment. An Australian study proposed that environmental countermeasures such as enhanced street lighting, medians, skid-resistant surfaces, and highly responsive pedestrian operated signals should be implemented in areas with high alcohol-related pedestrian-vehicle collisions [39].

Our findings did not implicate schools as a type of land use associated with Vancouver's pedestrian injury hotspots, despite the various other studies that have described the risk of pedestrian injury to children at or near schools as an important public health problem [21,40]. Road safety engineering is common in Vancouver on streets surrounding schools; particularly traffic-calming measures such as speed humps, road narrowing, and reduced speed limits designed to prevent pedestrian injuries among school children. These passive interventions can reduce the toll of paediatric pedestrian injury near schools through a reduction of speed and traffic volumes in sensitive areas [41]. Traffic-calming and environmental countermeasures should also be aggressively pursued in other parts of the city, especially in areas of elevated pedestrian use such as Vancouver's DTES and streets with a high density of alcohol serving establishments. There are fewer options available to calm traffic and improve pedestrian safety on arterial roads; however reducing the width of vehicle lanes can reduce the overall speed of vehicles on busy thoroughfares [42]. Also, a simple reduction of speed limits in high-risk areas is likely to be effective. Measures such as this, coupled with engineering modifications including medians or refuge islands, corner bulges, and controlled midblock crosswalks could be implemented with probable benefits for pedestrian safety.

### Limitations

This study has several limitations. The socio-demographic characteristics of the location of injury (average income, age of the population), and of the injured pedestrian (age, income, etc.) were not addressed. We focused on the contribution of the built-environment to pedestrian injury because much less is known about the relationship between roadway design and land-use type with pedestrian injury, compared with its social correlates. Also, it is possible that aspects of the built-environment not considered (or overlooked) in our analysis may be associated with pedestrian injury at these hotspot sites. For instance, we did not examine land use in detail nor did we account for weather or traffic volumes. Another limitation may be that the characteristics of a whole area could potentially have a greater effect on pedestrian injury than those of individual incident locations. For example, there is likely an area effect behind the clustering of multiple high-incident locations in the DTES. Also, while our designation of a pedestrian injury hotspot as a location with 5 or more incidents over the time period was done in order to set parameters on the scope of the analysis, there are no firmly established precedents in the literature regarding hotspot determination.

Another potential limitation of this research is the reliance on raw numbers of incidents rather than using a denominator population. We did this for several reasons. First, many of the incidents occurred in high traffic areas that were not particularly high population density regions (e.g. the DTES). Thus residential population density would not be a good indicator of pedestrian or road traffic. Second, many of the incident locations were not coincident with the home residence of the victims. Third, our chief focus was the examination of urban design that facilitates greater rates of injury.

## Conclusion

This study highlighted the disproportionate burden of pedestrian injury centred on the DTES area of Vancouver through undertaking a spatial analysis of pedestrian injury and subsequent environmental scan of hotspots. The environmental scan revealed that some important passive pedestrian safety countermeasures were only present at a minority of high-incident locations. Our findings support those of other studies which associate density of bars with pedestrian injury; however, there was no such association with schools. These results provide a foundation for extending pedestrian injury research as well as instituting passive intervention efforts. Future studies should analyze the effectiveness of built-environment modifications on reducing rates of pedestrian injury in areas such as those highlighted in this study.

## Competing interests

The authors declare that they have no competing interests.

## Authors' contributions

NS conceptualized and designed the study, participated in the environmental scan as well as in writing the paper. JC conducted the spatial analysis, participated in the environmental scan and contributed substantially to writing the paper. VC participated in the environmental scan and in writing the paper. SMH contributed to the study design and participated in the environmental scan.

## Pre-publication history

The pre-publication history for this paper can be accessed here:


